# Lung Nodule Malignancy Classification Integrating Deep and Radiomic Features in a Three-Way Attention-Based Fusion Module

**DOI:** 10.3390/jimaging11100360

**Published:** 2025-10-13

**Authors:** Sadaf Khademi, Shahin Heidarian, Parnian Afshar, Arash Mohammadi, Abdul Sidiqi, Elsie T. Nguyen, Balaji Ganeshan, Anastasia Oikonomou

**Affiliations:** 1 Concordia Institute for Information Systems Engineering, Montreal, QC H3G 1M8, Canada; sadaf.khademi@concordia.ca (S.K.); arash.mohammadi@concordia.ca (A.M.); 2 Department of Electrical and Computer Engineering, Concordia University, Montreal, QC H3G 1M8, Canada; 3 Department of Medical Imaging, Sunnybrook Health Sciences Centre, University of Toronto, Toronto, ON M4N 3M5, Canada; abdulwahab.sidiqi@ahs.ca; 4 Department of Medical Imaging, University Health Network, University of Toronto, Toronto, ON M5G 2N2, Canada; elsie.nguyen@uhn.ca; 5 Institute of Nuclear Medicine, University College London, 235 Euston Road, London NW1 2BU, UK; b.ganeshan@ucl.ac.uk

**Keywords:** lung cancer, malignancy classification, deep learning, attention fusion, auto-encoder, vision transformer

## Abstract

In this study, we propose a novel hybrid framework for assessing the invasiveness of an in-house dataset of 114 pathologically proven lung adenocarcinomas presenting as subsolid nodules on Computed Tomography (CT). Nodules were classified into group 1 (G1), which included atypical adenomatous hyperplasia, adenocarcinoma in situ, and minimally invasive adenocarcinomas, and group 2 (G2), which included invasive adenocarcinomas. Our approach includes a three-way Integration of Visual, Spatial, and Temporal features with Attention, referred to as I-VISTA, obtained from three processing algorithms designed based on Deep Learning (DL) and radiomic models, leading to a more comprehensive analysis of nodule variations. The aforementioned processing algorithms are arranged in the following three parallel paths: (i) The Shifted Window (SWin) Transformer path, which is a hierarchical vision Transformer that extracts nodules’ related spatial features; (ii) The Convolutional Auto-Encoder (CAE) Transformer path, which captures informative features related to inter-slice relations via a modified Transformer encoder architecture; and (iii) a 3D Radiomic-based path that collects quantitative features based on texture analysis of each nodule. Extracted feature sets are then passed through the Criss-Cross attention fusion module to discover the most informative feature patterns and classify nodules type. The experiments were evaluated based on a ten-fold cross-validation scheme. I-VISTA framework achieved the best performance of overall accuracy, sensitivity, and specificity (mean ± std) of 93.93 ± 6.80%, 92.66 ± 9.04%, and 94.99 ± 7.63% with an Area under the ROC Curve (AUC) of 0.93 ± 0.08 for lung nodule classification among ten folds. The hybrid framework integrating DL and hand-crafted 3D Radiomic model outperformed the standalone DL and hand-crafted 3D Radiomic model in differentiating G1 from G2 subsolid nodules identified on CT.

## 1. Introduction

Lung cancer remains the leading cause of death from cancers worldwide [1]. Non-Small-Cell Lung Carcinoma (NSCLC) is the most common type, accounting for approximately 85% of lung cancer cases [2]. Amongst NSCLC cases, lung adenocarcinoma is the most common histologic type [3,4]. Detection of lung cancer at an early stage can significantly reduce the mortality rate associated with this type of malignancy [5,6]. Early-stage manifestation of lung cancer is usually in the form of a pulmonary nodule. A pulmonary nodule is defined as well-defined or ill-defined rounded or irregular opacity with a diameter less than 3 cm. Pulmonary nodules are, typically, classified based on their density on Computed Tomography (CT) into three categories: (i) *Solid Nodules*, demonstrating a uniform soft tissue density; (ii) *Ground Glass Nodules (GGNs)*, characterized by a slightly high density through which vessels and bronchi can still be appreciated; and (iii) *Part-Solid Nodules*, comprising a varying density, with areas of both solid and ground glass density present at different locations [4]. Among these three types, part-solid nodules and GGNs (collectively referred to as SubSolid Nodules (SSN)) have been reported to have a higher risk of malignancy [7] and commonly represent adenocarcinoma spectrum disease lesions. According to the 2011 International Association for the Study of Lung Cancer classification, lung adenocarcinomas were categorized into 3 classes: pre-invasive lesions including Atypical Adenomatous Hyperplasia (AAH) and Adenocarcinoma In Situ (AIS), Minimally Invasive Adenocarcinoma (MIA), and Invasive Pulmonary Adenocarcinoma (IPA). Accurate classification of pulmonary nodule invasiveness is critical due to the favorable prognosis of the non-invasive nodules that may not need aggressive surgical treatment remaining under surveillance for a longer time and have a 90–100% of 5-year survival rate with surgical resection [4]. Specifically, pre-invasive lesions are characterized by a pure lepidic growth pattern, while minimally invasive lesions are defined as a predominant lepidic pattern with any histologic subtype other than a lepidic pattern. In early-stage disease, the prognostic significance of histologic subtypes becomes evident, where lepidic subtypes indicate a good prognosis and solid subtypes suggest a poor prognosis [8]. After initial diagnosis, a period of follow-up CT is a good method for differential diagnosis. However, if patients undergo surgical resection within a curative time window, they can be considered cured. This surgical curative time window is defined by pathological disease stages (including AIS and MIA stages) where the 5-year recurrence-free survival or disease-free survival reaches 100% after complete resection [9,10]. CT is the imaging modality of choice for the detection and characterization of pulmonary nodules [11]. Radiologists typically rely on morphologic and qualitative characteristics of nodules in CT scans to characterize nodules and determine if they are malignant or benign, such as shape, density, presence of speculations, calcification or fat density; however, this can be challenging and subjective, as there is often significant variability among observers. The gold standard for diagnosis of lung cancer depends on histological analysis, which is performed through invasive procedures such as an imaging-guided or bronchoscopic biopsy or surgical resection, for which not all patients may be eligible. Therefore, other non-invasive methods would be required as an alternative to biopsy-proven histology.

To address the above-mentioned issues, recently, there has been a significant surge of interest [12] in investigating the use of Artificial Intelligence (AI) to enhance accuracy of nodule invasiveness. Machine Learning (ML) and Deep Learning (DL) models are two important subfields of AI that are widely used in developing diagnosis algorithms. Currently, several algorithms for Computer-Aided Detection (CAD) systems have been proposed to act as a second pair of eyes for radiologists to assist them in differentiating subtypes of lung cancer. According to the type of input features, existing studies on nodule malignancy classification can be categorized into three main classes [13]: Hand-Crafted (HC) Radiomic, Deep Learning (DL) Radiomic, and Hybrid Models. HC Radiomic models use engineered features to describe nodule characteristics, but may miss critical information, limiting their accuracy [14]. DL Radiomic models automatically extract features using deep neural networks and can handle both 2D [15] and 3D [16] images, but they require large, annotated datasets, which are difficult to obtain. Hybrid Models [17], which are still in development, combine HC and DL features to enhance classification accuracy by leveraging the strengths of both approaches. This study focuses on developing a hybrid model to improve the diagnosis of lung nodules by integrating HC and DL Radiomic features.

Capitalizing on the above discussion, this study focuses on the third category, aiming to develop an innovative hybrid solution that combines HC and DL Radiomic features to provide improved performance and robustness in diagnosing the type of lung nodule. Before highlighting our contributions, first, we briefly outline the progress of DL-based solutions (particularly sequential models) for lung cancer diagnosis.

**Literature Review**: In order to balance the need for informative features and computational complexity, processing volumetric CT scans through sequential DL models can be a suitable approach. Transformer is a type of sequential model that was introduced by Vaswani et al. in 2017 and has since become a popular choice for processing sequential data, especially in Natural Language Processing (NLP) domain such as text categorization, machine translation, and question answering [18,19,20,21]. The Transformer architecture is built based on the attention concept that allows for the model to selectively focus on different parts of the input sequence when making predictions. Unlike traditional sequential models such as Recurrent Neural Networks (RNNs), Transformers do not require sequential processing of input data, making them more computationally efficient and easier for parallelization. For computer vision applications, a type of Transformer model was introduced as Vision Transformer (ViT) by Dosovitskiy et al. in 2020 [22]. In general, ViTs break the input image into a grid of patches, which are then treated as tokens in a sequence. Each patch is assigned an embedding vector that represents its features, and the sequence of these embedding vectors is processed by multiple Transformer blocks to extract high-level features and make predictions. This approach allows for ViT to learn spatial relationships between the image patches and capture global features of the image. Additionally, in attention-based models, the value of a pixel is derived from the whole image, which enables the model to attend to the most relevant patches. On the contrary, in convolutional models, the new value of a pixel depends on a limited receptive field of k×k neighboring pixels, which commonly disregard the non-local correlation and long-term dependencies within images, failing to capture inherent relations. ViTs have proven to be highly effective in the field of medical image analysis, showcasing successful applications in a diverse set of clinical tasks. These tasks encompass a wide range of functions, such as image segmentation, classification, detection, restoration, registration, and synthesis [23]. As our study focuses on the classification task, next, we will extensively explore and delve into this specific subfield.

Most researched and investigated Transformer-based approaches developed for medical imaging classification (according to disease type) can be divided into 3 main categories, COVID-19, tumor (lung, brain, breast, stomach), and retinal disease [24], with the highest proportion of research studies focusing on developing architectures for diagnosing COVID-19. However, in this short span of time, researchers have explored the various architectures of Transformer models to effectively classify lung tumors, some of which are presented independently and some in combination with other DL models. Recently, Barbouchi et al. [25] presented a Transformer-based deep neural network, called DETR Transformer, designed for the detection and classification of 3 types of lung carcinomas using PET/CT images. The combination of CT and PET scans enables a more detailed analysis of tumor characteristics, facilitating identification of tumor location and assessment of metabolic activity. The DETR Transformer model employs a Convolutional Neural Network (CNN) backbone to extract a condensed feature representation from PET/CT images. These features are subsequently fed into a Transformer encoder, which passes the output embeddings of the decoder section to a feed-forward neural network for class label prediction. Despite achieving an impressive accuracy of 96% in classifying histologic types of carcinomas using 270 image samples, the practicality of this model for routine scans and screening programs is limited. The main reason is the cost and availability of PET scans, which are not economically feasible for widespread use in many hospitals. Therefore, implementation of the DETR model in real-world clinical settings may face challenges due to the financial constraints associated with PET scans [25]. Later, a study by Sun et al. applied a Shifted-Window hierarchical Transformer, called SWin Transformer, on the LUNA16 dataset, including 888 low-dose lung CT images from Lung Image Database Consortium and Image Database Resource Initiative LIDC-IDRI dataset [26] for binary classification of nodules, and accomplished an accuracy of 82.3% for malignant/benign nodules. The observed classification accuracy indicates that utilizing CT scan images, in contrast to using PET/CT images as input [25], has limitations in effectively capturing tumor characteristics [27]. Several research studies have also incorporated 3D-based structures, such as the 3D Nodule detection Vision Transformer (3D-NodViT) [28] and the 3D Multi-Scale Vision Transformer (3D-MSViT) [29] architectures. These architectures utilize the capabilities of the SWin Transformer and have demonstrated superior classification results compared to previous models based on 2D and 3D CNNs, specifically for the LUNA16 dataset. While these models are less memory-intensive than convolutional models, they still necessitate more storage than 2D attention-based algorithms. Moreover, it should be mentioned that performance of the aforementioned models may be decreased in practical applications as the dataset used in these studies has a maximum slice thickness of 2.5 mm, and lung nodules are predominantly identified from CT scans obtained for various clinical purposes using routine standard or low-dose scanning protocols, which may involve non-thin slice thicknesses of up to 5 mm.

**Contributions**: Capitalizing on the above discussion and addressing the identified shortcomings of existing solutions, this paper proposes a novel hybrid lung nodule malignancy classification framework, referred to as the I-VISTA, which integrates DL and HC Radiomic features extracted via three parallel processing modules. In summary, the contributions of this study are as follows:

- The main contribution of this article is to indicate the superiority of combining HC and DL Radiomic features compared to stand-alone HC/DL models in improving the performance and robustness of diagnosing the type of lung nodule. This objective has been achieved by using a 3-way attention-based fusion module for the first time, to the best of our knowledge, integrating three independent feature sets to classify nodule’s invasiveness.
- Each of the constituent feature extraction paths of the proposed I-VISTA framework concentrates on a specific domain to capture local and global evidence of invasive nodules. More specifically, the I-VISTA takes the inherent three-dimensional characteristics of non-thin CT sequences. It accomplishes this by simultaneously incorporating the temporal (between-slice) and spatial (within-slice) variances of CT slices with quantitative features of nodules in an attention-based fusion center.
- The fusion module is constructed based on a Criss-Cross Attention (CCA) framework that combines three feature sets effectively providing complementary information about each nodule considering its spatial, temporal, and visual attributes extracted from non-thin lung CT scans of 114 pathologically approved nodules. The spatial and temporal characteristics of each nodule were extracted with Transformer-based models and fed to the CCA module, along with nodule HC Radiomic features. The CCA module can well-learn the global contextual information among three sets of features and pay attention to strong discriminative features to distinguish the type of nodule. The proposed integration approach allows for the I-VISTA model to capture the interactions and dependencies between three feature sets, resulting in a higher level of performance than either set can achieve alone.

## 2. Materials and Methods

This retrospective study was approved by the Research Ethics Board (REB) of a single institution (study ID: 379-2015), and patient consent was waived due to the retrospective nature of the study. Furthermore, all methods were carried out according to relevant guidelines and regulations. The REB determined that an informed consent form was not required for this study.

**Figure 1:**
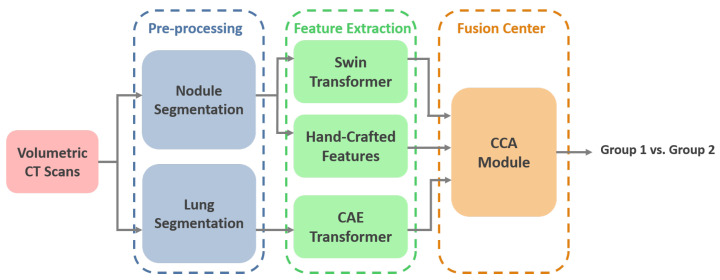
I-VISTA model architecture. Volumetric lung CT scans undergo segmentation, followed by the extraction of visual, spatial, and temporal features. The integration of these features is achieved through a criss-cross attention module. SWin: Shifted Window, CAE: Convolutional Auto-Encoder, CCA: Criss-Cross Attention.

The overall schematic of the I-VISTA framework is shown in Figure 1. The proposed model aims to improve lung nodule invasiveness classification accuracy using a combination of DL and HC Radiomic features. To achieve this, our model presents three parallel processing paths that extract the aforementioned features from CT images followed by a 3-way attention module to integrate the generated features and perform classification. In other words, two DL models, each concentrating on a specific variation field of nodules, were developed and combined with HC Radiomic features to improve the overall performance of nodule malignancy classification. In what follows, we will present details of the utilized dataset for the model assessment, along with the pre-processing steps required for each of the three processing paths. Afterwards, we will introduce each of our triple-path feature extraction procedures to develop desired inputs for the integration module. Finally, we will describe the feature fusion and classification mechanisms incorporated into the 3-way attention-based module.

### 2.1. Data Description

**Figure 2:**
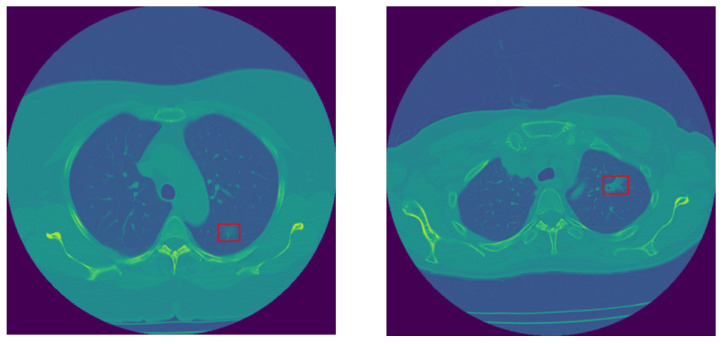
Sample non-invasive (**left**) and invasive (**right**) lung nodules.

In this study, we use the in-house dataset initially introduced in [30], and include five additional cases obtained from the same institution. This dataset contains volumetric chest CTs (1–3 mm slice thickness) of 114 SSNs, which were biopsy-proven adenocarcinoma spectrum disease lesions. The number of slices varies among patients; however, the collective count totals 925 across all cases. SSNs were categorized into two groups according to their pathological findings and the similarity in their survival rates, as follows: The low-risk group 1 (G1) included 28 pre-invasive lesions (AAH and AIS) and 30 minimally invasive nodules, totaling 58 low-risk lesions, and the high-risk group 2 (G2) included 56 invasive adenocarcinomas. All nodules were surgically resected. CT scans were acquired without contrast medium administration in one of the 2 scanners of a single institution (Aquilion One 64 and 320 detector row CT, Canon Medical Systems, Otawara, Japan). The technical parameters used were 100 to 135 kVp, 80 to 120 mAs, 1–3 mm slice reconstruction, gantry rotation time 0.35 s, and FOV 35–40 cm [30]. The last CT obtained before the resection date was chosen for the purpose of this research study. Examples of invasive and non-invasive nodules are shown in Figure 2.

### 2.2. Pre-Processing

For each patient, all the CT slices (originally sized 512 × 512 pixels) in which the nodule of interest was visible were identified and selected by an expert thoracic radiologist with 19 years of experience who was blinded to the pathology results. The selected slices were then passed in parallel through two different pre-processing stages to prepare the input structure for each feature-extraction path. These two pre-processing paths analyze each slice in large and small scales. One of them focuses on the segmentation of the lung parenchyma required for temporal variations, while the other focuses on the nodule segmentation desired for extracting HC Radiomic features and spatial variations of nodules.

#### 2.2.1. Small-Scale: 3 Dimensional Nodule Segmentation

An expert thoracic radiologist with 19 years of experience and a 3-year medical student segmented a volumetric region of interest using the commercially available research software TexRAD 3.9 (Feedback Medical Ltd., London, UK). The free-hand contouring tool was applied at all the CT slices where the nodule was visible, followed by manual correction whenever necessary. This software is widely used in medical imaging and has been described in numerous oncologic studies [31,32,33].

#### 2.2.2. Large-Scale: Lung Segmentation

**Figure 3:**
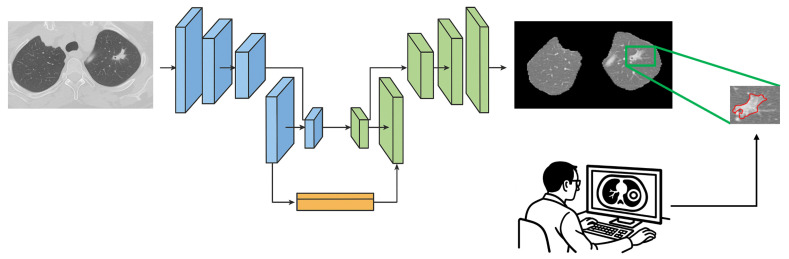
Sample of lung parenchyma (**left**) and nodule (**right**) segmented area. The red contour outlines the segmented region of interest.

In order to remove distracting components from CT images, lung parenchyma was segmented from the whole CT slice by adopting a U-Net-based model trained on the R-231 dataset, which includes scans acquired with 22 different combinations of scanner manufacturer, convolution kernel, and slice thickness. The segmented lung regions were subsequently resized to 256 × 256 pixels to ensure computational efficiency and consistency across patients before being used as input to the DL model [34]. The concept of U-Net was initially introduced by Ronneberger et al. for biomedical image segmentation [35]. The U-Net name originates from its U-shaped architecture consisting of an encoder and a decoder. The encoder component is a standard CNN that extracts features from the input image, while the decoder includes upsampling layers to reconstruct the image at its original resolution and generates a segmentation mask. The U-Net architecture has been effectively adopted in various medical image segmentation tasks, including segmenting the brain, lungs, and liver [36,37,38]. Figure 3 illustrates a representation of lung parenchyma and nodule segmented area.

### 2.3. Deep-Learning Radiomic Features

**Figure 4:**
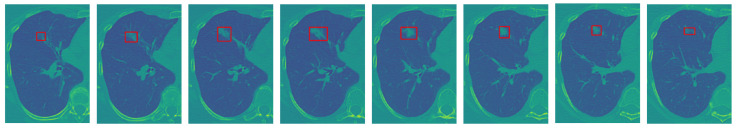
An illustrative example to clarify the rationale behind integrating temporal and spatial analysis within our framework (the red frame highlights the nodule region of interest): sequence of sample slices in a CT volume of a patient illustrating nodule’s location, size, and shape variation.

Given the fact that volumetric CT sequences are inherently 3D in nature, the I-VISTA model is capable of jointly analyzing the temporal and spatial features of volumetric CT scans with much less computational complexity compared to 3D DL models. Two out of three paths of the proposed framework were built based on DL models and developed to extract inter-slice (temporal) and within-slice (spatial) variations of the nodule in a volumetric CT scan using the Transformer architecture [18]. As depicted in Figure 4, by focusing on the red rectangle region of interest, it becomes evident that each slice of a volumetric CT scan presents a unique view of the nodule, illustrating its shape, size, and location variations. This highlights the necessity of integrating both temporal and spatial viewpoints implemented by two DL models, the SWin and Convolutional Auto-Encoder (CAE) Transformer. The SWin-Transformer architecture prioritizes the spatial characteristics of the nodule, addressing its smaller size relative to the lung lesion. In contrast, the CAE-Transformer focuses on capturing the temporal variations in nodule shape and location across slices. Next, we provide a detailed structure of each of the three constituent modules of the proposed I-VISTA framework.

#### 2.3.1. Learning Spatial Variations: (Supervised) SWin-Transformer

**Figure 5:**
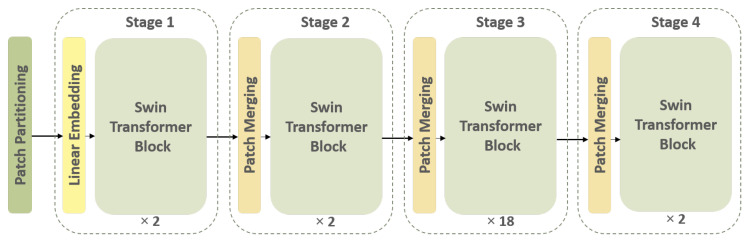
Overall architecture of the SWin Transformer. The input image undergoes patch partitioning, followed by hierarchical processing through Transformer blocks.

The first feature extraction path of the I-VISTA framework is developed based on a SWin Transformer [39], which is a DL model utilizing Transformers in a hierarchical fashion to identify relevant informative spatial characteristics within patches of nodules. The SWin Transformer design is constructed according to the ViT architecture [22], but it overcomes two major limitations encountered in previous ViT-based models. Specifically, the SWin Transformer deals with the challenges of computational complexity and fixed-scale patches by incorporating hierarchical feature maps and a shifted-window attention mechanism. Further details are provided in the Appendix A of this paper. As can be observed from the overall layout of the SWin Transformer in Figure 5, it has four processing stages with two main repetitive parts in each stage, which are the “Patch Merging” and “SWin Transformer” blocks.

Initially, the input image is partitioned into 4×4 patches. In the first stage, the feature dimension of each patch is projected into 128 using a linear embedding layer. Afterwards, the SWin Transformer blocks are applied to each patch feature vector. One of the main characteristics of the SWin Transformer is its ability to create a hierarchical feature representation of the input data, which is accomplished by the patch-merging technique. The resulting feature maps generated for each patch are merged together through this operation to produce a single high-level representation of the input signal. The patch merging operation concatenates features of neighboring patches into a unified representation. This process enables the model to capture both local and global information from the input image.

When it comes to processing volumetric CT scans, each slice provides a specific view of the nodule, which, if seen consecutively, illustrates the nodule region changes around a point. This point and its surrounding pixels are the key important pixels that the SWin Transformer must find, paying attention to its spatial variations. In order to acquire this type of variation, we implemented the SWin-B architecture, which consists of 2, 2, 18, and 2 layers for stages 1 to 4, respectively. The feature vectors obtained from stage 4 for each subject’s slices are utilized as input to a Global Max Pooling (GMP) layer. The GMP layer consolidates the spatial variations of nodules present in images of each subject, encapsulate the most significant features across all nodule slices [40]. The I-VISTA model is a fine-tuned version of a pre-trained SWin-B Transformer trained on the ImageNet-21k dataset [41] with an input image size of 224×224 and window size of 7×7. The model inputs were nodule patches cropped from CT slices based on the coordinates obtained from the small-scale pre-processing step for each SSN and zero-padded to (224, 224) pixels to have a fixed-size input for the SWin Transformer model. It is relevant to point out that attention-based models can selectively attend to relevant regions in the input. Given that the majority of the image is filled with zeros, the model will assign low attention weights to the zero-padded regions.

#### 2.3.2. Learning Temporal Variations: (Unsupervised) CAE-Transformer

The second processing path of our framework includes a Convolutional Auto-Encoder (CAE) module followed by a Transformer encoder designed to detect inter-slice variations of nodules. CAE is considered a type of neural network responsible for providing a compact representation of each slice in an unsupervised fashion [42]. CAE architecture’s main components are the encoder and decoder convolutional-based networks. The encoder network generates a lower-dimensional representation of the input data. On the other hand, the decoder network learns how to reconstruct the original input from the encoded features. The configuration of layers in our CAE model consists of 5 convolution layers (filter dimensions of 16, 32, 64, 128, and 256), each followed by a max-pooling layer (kernel size of 2×2) fed into a dense layer of size 256. The primary objective of the CAE model is to minimize the discrepancy between the original input data and its reconstructed version by means of Mean Squared Error (MSE), which leads to a more concise, informative representation of input data. To familiarize the overall model with a diverse range of lung cancer imaging data, the CAE module is initially pre-trained on the LIDC-IDRI dataset [26].

**Figure 6:**
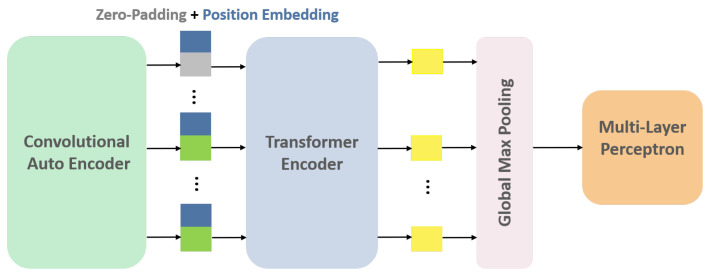
CAE-Transformer architecture. This configuration featuring a Convolutional Auto-Encoder (CAE) module followed by a Transformer encoder is designed for the detection of inter-slice variations in the nodule.

In this study, CAE is considered to be the embedding layer of Transformer architecture to project the input data into a lower-dimensional space with high-level information. The original embedding layer typically consists of a linear projection layer responsible for this task. To capture temporal variations between different slices available for the nodule, each slice acts as a single patch. In other words, the positional encoding step, which adds information about the spatial location of each patch, alternatively preserves slice position ordering in the CT volume. It should be noted that the number of slices showing a nodule varies between different individuals, ranging from 2 to 25 slices per nodule. In order to ensure the consistency of our input data, we have chosen to use the maximum number of slices (i.e., 25 slices) and padded the input-embedded feature map sequences with zeros. This padding ensures that all sequences provided by the CAE module for each patient have the same dimensions. After applying 3 stacks of Transformer encoders to the input instances (slices), the resulting outputs are fed into a GMP layer. The GMP layer selects the maximum value from each feature map, producing a fixed-length vector. This vector is then passed through a Multi-Layer Perceptron (MLP) layer with 32 neurons to produce the final feature vector (Figure 6). More detailed information is available in the Appendix A of this paper.

### 2.4. 3D Radiomic Features

The third processing path of our model focuses on acquiring a set of quantitative features obtained from the texture analysis of each nodule, known as HC Radiomic features. For each of the 114 nodules, we conducted a volumetric 3D segmentation and extracted six biologically meaningful radiomic features using a commercially available research software TexRAD (Feedback Medical Ltd., UK), employing six Spatial-Scale Filter (SSF) values. SSF = 0 corresponds to no filtration (conventional image), SSF = 2 mm represents fine-texture scale, SSF = 3, 4, 5 mm represents medium-texture scale, and SSF = 6 mm represents coarse-texture scale. SSFs are Laplacian of Gaussian (LoG) band-pass filters. The LoG filter combines Gaussian smoothing with Laplacian edge detection, enabling feature extraction at multiple spatial scales. Initially, Gaussian filtering reduces noise, followed by the Laplacian operator to enhance edges, allowing for the isolation of texture patterns at different scales. The Gaussian function is defined as

(1) $$G\left(x,y\right)=\frac{1}{2\pi \sigma^{2}}\exp\left(-\frac{x^{2}+y^{2}}{2\sigma^{2}}\right),$$

where (x,y) denotes a pixel coordinate and σ controls the extent of smoothing. The Laplacian operator applied to G(x,y) is

(2) $$\mathrm{Laplacian}\left(x,y\right)=\frac{\partial^{2}G\left(x,y\right)}{\partial x^{2}}+\frac{\partial^{2}G\left(x,y\right)}{\partial y^{2}}.$$

Substituting G(x,y) into the Laplacian gives the following LoG function:

(3) $$\mathrm{LoG}\left(x,y\right)=-\frac{1}{\pi \sigma^{4}}\left(1-\frac{x^{2}+y^{2}}{2\sigma^{2}}\right)\exp\left(-\frac{x^{2}+y^{2}}{2\sigma^{2}}\right).$$

The parameter σ determines the spatial scale of the texture detection. Larger σ values yield stronger smoothing and emphasize broader, low-frequency structures, while smaller σ values highlight fine, high-frequency textures. In our framework, σ values of 2, 3, 4, 5, and 6 were applied. Following filtration, the software automatically computes histogram and texture features, including Mean, Standard Deviation (SD), Entropy, Mean of Positive Pixels (MPP), Skewness, and Kurtosis [32,43]. Consequently, we acquire six radiomic features for each filter scale, forming a comprehensive 3D radiomic feature set across all nodules. In alignment with the approach taken for other feature sets, we adopt the strategy of flattening the 3D Radiomic feature array, yielding a standardized set of 36 radiomic features for each nodule.

### 2.5. Fusion Module: Features Integration via Criss-Cross Attention (CCA)

**Figure 7:**
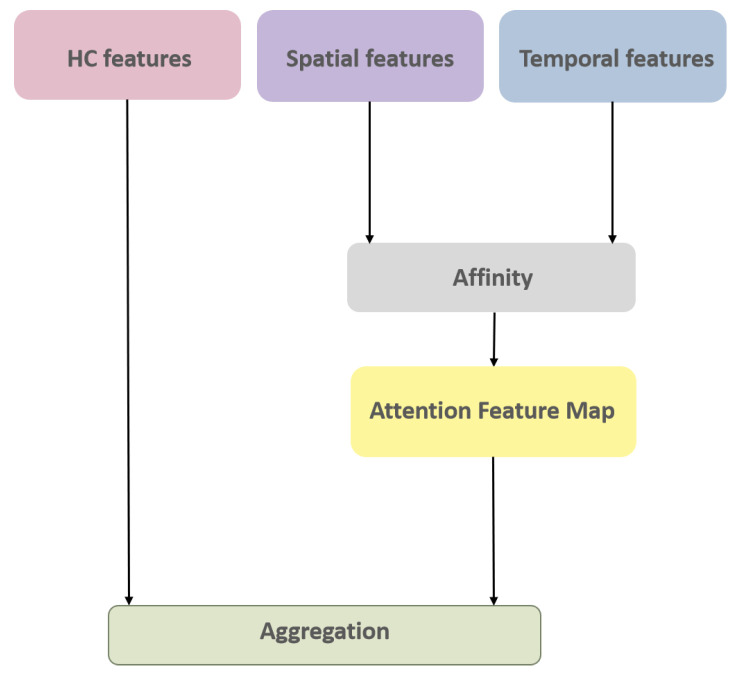
CCA module architecture.

Once the aforementioned 3 sets of feature maps (incorporating spatial and temporal variations of nodules and their HC Radiomic features) have been extracted, the next phase involves the appropriate fusion (integration) of these features to predict the invasiveness of each nodule. The I-VISTA framework performs information fusion by applying a three-way attention mechanism known as CCA [44], which was recently introduced by Huang et al.; however, we used this specifically for the task of image semantic segmentation. Here, we re-tasked the CCA toward the fusion of feature maps extracted via each of the three paths of the I-VISTA framework. The designed CCA is an extension of the self-attention mechanism to aggregate information across different spatial scales in an image using cross-scale feature interactions (horizontal and vertical directions). Figure 7 illustrates a block diagram of the CCA module, which involves two main operations, i.e., affinity and aggregation.

The affinity operation measures the similarity between the *Q* and *K* feature maps in horizontal and vertical pathways. In other words, this operation calculates the degree of correlation ($d_{i,u}$) at position *u* between each feature vector in the $Q_{u}$ feature map of size H×W and other feature vectors in *K*, which are in the same row or column ($\Omega_{i,u}$) with the position where i=[0,…,H+W−1], i.e.,

(4) $$d_{i,u}=Q_{u}\Omega_{i,u}^{T}.$$

The attention feature map ($A_{i,u}$) is then obtained by applying a softmax layer on the output of the affinity operation. The next step includes the aggregation of the attention map and *V* vector as the third feature map. This was accomplished by a weighted sum operation for each feature vector position, where the attention map plays the role of the weight matrix multiplied by a set of feature vectors in *V*, which are in the same row or column ($\Phi_{i,u}$) with that position, i.e.,

(5) $$H_{u}=\sum_{i=0}^{H+W-1}A_{i,u}\Phi_{i,u}.$$

The original study [44] applied 1×1 convolutional filters to generate feature maps of the input image. In this study, however, we used feature maps produced in parallel processing paths of our model to concentrate on the most informative characteristics in predicting nodule invasiveness. We projected all our feature maps’ dimensions to an identical dimension of 100 and then copied (passed) radiomic, spatial, and temporal feature maps to *Q*, *K*, and *V* vectors of CCA, respectively. The final integrated feature map is then utilized as the input for the classification of nodule invasiveness by applying a dense layer with two neurons and a softmax as its activation function.

## 3. Results

In this section, we comprehensively analyze and evaluate different aspects of the proposed I-VISTA framework using a ten-fold cross-validation technique. All experiments were trained on an NVIDIA GeForce RTX 3090 GPU, with 32 GB RAM and an AMD Ryzen 9 3900X 12-core processor. On average, the training times for the individual stages of the proposed architecture were as follows: approximately 90 s for the CAE stage, 500 s for the SWin Transformer stage, and 2 s for the CCA module. DL-based models in parallel paths were fine-tuned on the basis of independent pre-trained models (Table 1). To ensure effective training, careful selection of hyperparameters was crucial, particularly the learning rate. Learning rate is a key hyperparameter that affects model convergence; large learning rates during fine-tuning may cause catastrophic forgetting of pre-trained knowledge, whereas small learning rates for models trained from scratch may result in slow convergence or underfitting. In our framework, different learning rates were chosen for each component of the framework based on its role and initialization state to balance stability and performance. The CAE model was initially trained on the LIDC-IDRI dataset mentioned earlier. Afterward, the best model was selected based on its performance in a hold-out fashion (80–20%) and further fine-tuned on the in-house dataset. During the fine-tuning process, only the middle Fully Connected (FC) layer and its preceding and succeeding convolution layers were trained, while the remaining layers were frozen. The features generated by the fine-tuned CAE model were then fed to the Transformer encoder. The pre-trained SWin Transformer was fine-tuned on our in-house dataset. Then, two DL-based feature sets along with HC Radiomic features were passed through the CCA module to find the most significant features for classifying nodule type. In the entire process, binary cross-entropy was used as the loss function, and an early-stopping strategy was implemented along with dropout layers to mitigate the risk of overfitting. The code and the in-house dataset will be made available upon reasonable request to ensure the reproducibility of the results.

To evaluate the classification performance, we employed the following metrics: Accuracy, Sensitivity, Specificity, Precision, and F1 Score. Let TP, TN, FP, and FN denote the number of true positives, true negatives, false positives, and false negatives, respectively.

- **Accuracy** measures the proportion of correctly classified samples:
  (6) $$\mathrm{Accuracy}=\frac{TP+TN}{TP+TN+FP+FN}$$
- **Sensitivity** measures the proportion of actual positives correctly identified:
  (7) $$\mathrm{Sensitivity}=\frac{TP}{TP+FN}$$
- **Specificity** measures the proportion of actual negatives correctly identified:
  (8) $$\mathrm{Specificity}=\frac{TN}{TN+FP}$$
- **Precision** measures the proportion of predicted positives that are correct:
  (9) $$\mathrm{Precision}=\frac{TP}{TP+FP}$$
- **F1 Score** measures the harmonic mean of Precision and Recall:
  (10) $$F1\;\mathrm{Score}=2\cdot \frac{\mathrm{Precision}\cdot \mathrm{Recall}}{\mathrm{Precision}+\mathrm{Recall}}$$

In the following paragraphs, the results are presented to serve the purpose of comparing different classification techniques. These results have been organized into three distinct sections, namely HC Radiomic-based, DL Radiomic-based, and I-VISTA Hybrid model, each dedicated to evaluating a specific type of feature-extraction technique. By dividing the analysis into these sections, we aim to provide a comprehensive understanding of the performance and characteristics of each technique.

### 3.1. HC and DL Radiomic-Based Models

**Figure 8:**
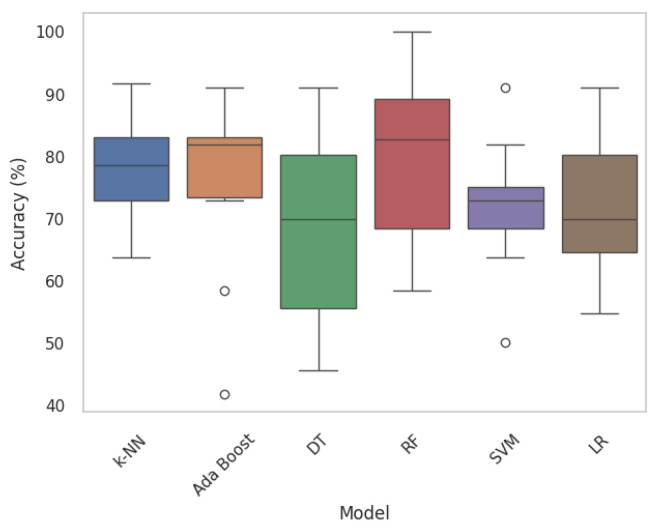
Boxplots of traditional ML models. Each boxplot provides insights into the variability and central tendencies of model performance. Circles mark outlier data points.

In this section, nodule classification is initially accomplished using HC Radiomic features as the input to traditional ML models, which are established based on a set of statistical principles. A performance comparison table among the most common ML classification algorithms, namely Ada Boost, Random Forest (RF), k-Nearest Neighbors (k-NN), Decision Tree (DT), and Logistic Regression (LR) across ten folds is provided in Table 2. Traditional ML baselines were implemented using PyCaret library with default scikit-learn hyperparameters. Moreover, Figure 8 displays boxplots representing the model’s classification accuracy values, offering valuable insights into the distribution of accuracy across different folds. Following that, we executed a comparative analysis between proposed DL-Radiomic models, SWin-Transformer capturing spatial patterns in nodule patch, and CAE-Transformer focusing on temporal patterns of nodule in lung parenchyma in processing varying scales of the input data. Results are shown in Table 3.

### 3.2. I-VISTA Hybrid Model

**Figure 9:**
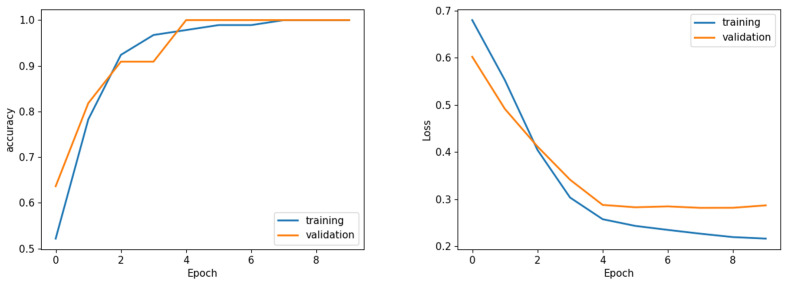
Sample learning curve (Fold 1) accuracy (**left**) and loss (**right**) analysis. Sample of loss/accuracy convergence during training CCA (Fold 1).

**Figure 10:**
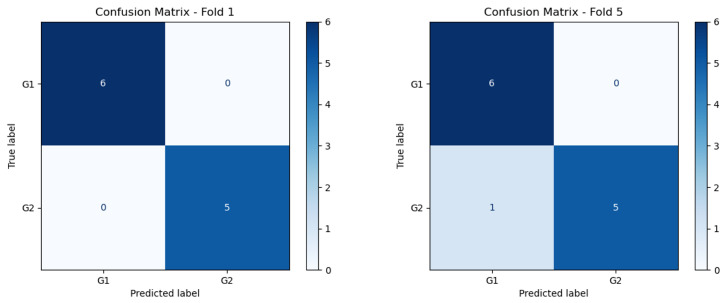
Confusion matrix of Folds 1 and 5 of the best performing model.

**Figure 11:**
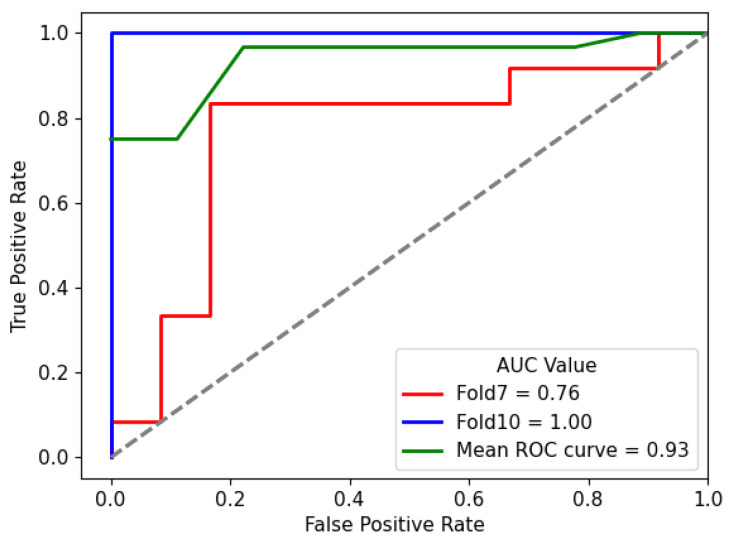
Mean ROC curve of I-VISTA framework variation among ten folds for the best performing model. The dotted diagonal line represents the performance of a random classifier (AUC = 0.5).

The results presented in this section underscore the effectiveness of the proposed hybrid model in accurately classifying lung nodule types. The combination of the DL and HC Radiomic models, along with an attention-based fusion module, provides promising results for enhancing the diagnostic capabilities of radiologists and assisting in the early detection of invasive pulmonary nodules. The classification outcomes for each fold were subsequently evaluated according to optimized weights obtained during the training phase. Sample graphical representations of I-VISTA learning curves are illustrated in Figure 9. Table 4 provides a summary of the model’s performance across various folds, offering insights into its effectiveness in classifying nodules. In addition, confusion matrices for Folds 1 and 5, as examples, are provided to demonstrate the classification performance across different folds Figure 10. In Figure 11, we provided the mean Receiver Operating Characteristic (ROC) curve of our hybrid model, alongside its variability spanning from the worst to the best outcomes. The ROC curve visualizes the trade-off between the true positive rate (sensitivity) and false positive rate (1—specificity) at different classification thresholds. The Area Under the Curve (AUC) is computed as the integral of the ROC curve, providing a single summary measure of the model’s discriminatory ability, with values closer to 1 indicating better performance. This range of outcomes yielded an AUC value of 0.93±0.08. The mean ROC curve visually represents the model’s overall discriminatory power, while the variation depicted highlights the extent of performance fluctuations across different folds.

## 4. Discussion

First, we focus on evaluating the results of 3D HC Radiomic features being fed to classical ML models (Table 2 and Figure 8). In this regard, the most effective classical ML classifiers are initially identified [45] and their hyperparameters are refined through a grid-search fine-tuning process. According to the classification results of the HC Radiomic-based models presented in Table 2, processing quantitative visual features of nodules by traditional ML models yields considerably inferior performance in comparison to the proposed hybrid framework. Based on the fine-tuned performance results, it can be observed that RF outperforms other statistical ML models in terms of being the most discriminative classifier. Our findings are consistent with the conclusion drawn from a recent study [45], which compared a DL-based model (multiple-scale residual network) to well-known traditional ML models (KNN, DT, RF, SVM, and Ada Boost), indicating that DL-based models hold the potential to outperform traditional ML methods in lung nodule classification tasks. When comparing our methodology in extracting 3D HC Radiomic-based features with the existing literature [46,47,48], its effectiveness stands out. In other words, existing studies typically start with a wide range of radiomic features, often in scale of thousands, followed by complex feature-selection techniques such as Least Absolute Shrinkage and Selection Operator (LASSO). Our methodology is distinct in its focus on leveraging a significantly smaller subset of radiomic features, specifically six biologically meaningful ones, for lung nodule invasiveness classification. While employing a smaller number of features may seem counterintuitive compared to the more traditional approach of utilizing a larger feature space, it is important to note that our feature selection process is guided by a thorough understanding of the underlying biological specifications driving nodule invasiveness. This targeted approach not only reduces the dimensionality of our feature space, but also mitigates the risk of overfitting and enhances the generalizability of our model.

Although models built upon HC Radiomic features have undoubtedly contributed valuable insights, they often struggle to capture the full complexity inherent in the data due to the constraints imposed by predefined calculations. This starkly highlights the contrast between the efficacy of combining DL and HC features to overcome the limitations posed by solely relying on HC Radiomic-based approaches.

Conversely, individual DL models reveal their limitations in effectively addressing the lung nodule classification challenge. This deficiency is evident in the outcomes detailed in Table 3. However, a closer observation shows the superior performance of the SWin-Transformer model compared to the CAE-Transformer. These classification outcomes suggest that smaller-scale analysis holds the potential to outperform larger-scale analysis. This advantage stems from its focus on specific image regions due to challenging nature of the task related to the small size of nodules compared to the lung parenchyma [49]. However, to achieve a more comprehensive understanding of nodule variations, a combination of both approaches is essential. In other words, spatial and temporal analyses complement each other by providing a more holistic perspective of nodules. The multi-view nature of nodules has been investigated in numerous research studies by employing a combination of multiple 3D DL models to analyze CT scans comprehensively [50] or via utilization of Long Short-Term Memory (LSTM) networks [51] to capture long-term dependencies in sequential data. However, a notable drawback of these approaches lies in their computational cost compared to attention-based 2D processing models. Moreover, the attention mechanisms integrated into our model allow for the selective weighting of input features, enabling the model to prioritize salient information during the analysis process.

The learning curves shown in Figure 9 demonstrate the stability of the proposed I-VISTA model, particularly in the context of analyzing loss values, which was a critical factor in choosing model weights. Training and validation accuracy and loss graphs provide insight into the learning dynamics of the model. Training metrics are computed on the dataset used to update model weights, while validation metrics are computed on unseen data to assess generalization. These graphs are crucial for detecting issues such as overfitting, underfitting, or unstable training. The convergence of training and validation curves towards lower loss values indicates the significance of the early-stopping strategy, effectively preventing model from overfitting.

With regards to the results shown in Table 4, in addition to the five folds (fold 1, 4, 6, 9, and 10) that exhibited the best performance metrics, it is noteworthy that the overall standard deviation of metrics is notably smaller than HC/DL Radiomic models. This reduction in variability underscores the stability and consistency of our model’s performance across different combinations of data.

We performed a statistical analysis to evaluate the significance of the differences between folds. Statistical analysis of the ten-fold cross-validation performance data demonstrates no statistically significant differences between folds (Kruskal–Wallis and Friedman tests both *p* > 0.05). Despite an 18.19% performance range from 81.81% to 100%, the coefficient of variation is only 7.23%, indicating excellent model stability well below the 10% threshold for acceptable variability. Overall, these results confirm the stability and generalization capability of the model across folds.

Complementarily, the compact range of AUC values in Figure 11, centered around 0.93, signifies the consistent and favorable performance of the hybrid model in accurately distinguishing between different nodules.

By bridging the gap between HC Radiomic and cutting-edge DL Radiomic features in an attention-based fusion module, CCA, the proposed I-VISTA model not only demonstrates its efficacy, but also exemplifies a paradigm shift in medical image analysis. In our framework, CCA served as the feature combinator, contrasting with other fusion prediction studies that mostly concatenate feature sets and employ linear functions such as LR [17], and FC layers [52]. Particularly, LR and FC layers provide a straightforward interpretation of feature coefficients, assuming linear relationships between features and the target variable, while CCA’s flexibility allows for the model to adapt to diverse and non-linear patterns of the data. Additionally, CCA, having the capacity for nonlinear combinations of features, dynamically attends to various parts of the input space, capturing intricate relationships and dependencies within heterogeneous data sources and offering insights into feature importance through attention weights.

Generally speaking, it is difficult to directly compare our study with previous works, as models are developed based on different datasets. However, Table 5 represents the overall classification performance of our reference study [30] evaluated for ten folds. As is evident, the performance metrics of the I-VISTA model exhibit higher values compared to [30], which is an HC Radiomic-based model extracting 2D Radiomic features obtained from the geometric and statistical characteristics of CT attenuation parameters of each nodule. In this table, accuracy and AUC metrics are presented with a 95% Confidence Interval (CI) to describe the uncertainty level of models. Of note, the narrower range of CI holds substantial benefits. A narrower CI indicates a higher level of precision and confidence in the estimated performance, minimizing the variability of the results and enhancing the reliability of our findings. This tighter range underscores the consistency and robustness of the proposed model, strengthening its potential for accurate lung nodule invasiveness classification.

Our study has several limitations. First, this is a retrospective study, with the inherent limitations of the retrospective nature of a study being that it introduces a selection bias in the cohort, as only surgically resected nodules were included. Second, although the proposed hybrid model demonstrates strong performance, the inherent black-box nature of DL models raises challenges in interpreting their decision-making process. Ensuring the clinical meaningfulness and transparency of the model’s decisions in an interpretable manner for radiologists remains an ongoing area of research. Third, this was a small cohort of cases from a single institution. A more extensive and diverse pathologically proven dataset is needed to allow for a more comprehensive assessment of the model’s capabilities. In addition to that, due to the small number of cases, we focused on a binary classification, which may not reflect the full range of complexity of the adenocarcinoma spectrum disease lesions. Furthermore, although the CT studies were performed in only 2 different CT scanners in a single institution, slightly different protocols were used, resulting in a range of slice thickness between 1 and 3 mm. However, this represents a more realistic routine clinical practice, increasing the external applicability of our hybrid model due to this technical variability. Lastly, external validation is lacking in our study; however, the ten-fold cross-validation is considered a robust method of internal validation. These limitations highlight avenues for future research to refine and expand our model’s applicability and interoperability.

## 5. Conclusions

In conclusion, in this study, we introduce a novel hybrid three-way attention-based fusion model referred to as I-VISTA, designed for the classification of lung nodule invasiveness in CT scans. The proposed I-VISTA framework successfully accomplishes this objective by integrating spatial, temporal, and visual feature sets derived from the analysis of slices within a CT volume through its three distinct processing parallel paths. These pathways encompass a combination of two deep-learning feature extraction algorithms and one hand-crafted approach, collectively enabling the framework to comprehensively capture and analyze the relevant information. Fusion of these features takes place within a Criss-Cross Attention module, allowing for the model to identify and prioritize the most meaningful patterns from 3 input feature sets. Analysis of the proposed hybrid model, in comparison to the stand-alone utilization of deep and hand-crafted features, demonstrates a higher level of performance consistency. The model’s ability to unveil hidden patterns and intricate details within medical images complements the expertise of radiologists, potentially leading to more accurate diagnoses and tailored treatment strategies. Indeed, the model has the capacity to significantly aid radiologists in discerning factors and variables that might remain elusive to the human eye.

## Figures and Tables

**Table 1:** Hyperparameter settings for each training stage of the I-VISTA framework.

Model/Stage	Optimizer	Learning Rate	Epochs	Batch Size	Weight Decay	Patience	Drop-Out
CAE/Pre-training	Adam	$1\times 10^{-4}$	200	128	NA	NA	NA
CAE/Fine-tuning	Adam	$1\times 10^{-6}$	50	64	NA	NA	NA
CAE-Transformer/Training	Adam	$1\times 10^{-3}$	200	64	NA	NA	0.3
Swin Transformer/Training	AdamW	$1\times 10^{-5}$	50	1	0.05	10	0.1
CCA ModuleTraining	AdamW	$1\times 10^{-3}$	20	8	0.05	5	NA

**Table 2:** Patient-level classification performance (mean ± std) of traditional ML models applied on HC Radiomic features under minimal randomness over ten folds.

Model	Accuracy (%)	Sensitivity (%)	Specificity (%)	AUC
DT	67.72 ± 15.22	63.66 ± 25.45	73.00 ± 20.93	0.68 ± 0.15
LR	71.89 ± 11.01	70.00 ± 27.26	74.80 ± 15.68	0.86 ± 0.09
SVM	72.12 ± 10.83	74.00 ± 34.52	70.47 ± 23.75	0.72 ± 0.11
Ada Boost	75.83 ± 15.28	72.00 ± 21.09	70.99 ± 32.81	0.84 ± 0.16
k-NN	78.03 ± 9.48	**80.33 ± 16.28**	76.91 ± 20.09	0.87 ± 0.12
RF	**79.16 ± 13.43**	73.33 ± 25.53	**84.66 ± 18.40**	**0.88 ± 0.11**

**Table 3:** Patient-level classification performance (mean±std) of I-VISTA and its components under minimal randomness over ten folds.

Model	Accuracy (%)	Sensitivity (%)	Specificity (%)	AUC
CAE-Transformer	69.46 ± 16.42	64.33 ± 21.45	74.66 ± 19.95	0.71 ± 0.20
SWin-Transformer	78.10 ± 12.45	76.66 ± 21.80	79.66 ± 17.91	0.80 ± 0.15
I-VISTA	**87.87 ± 11.02**	**82.66 ± 21.69**	**93.33 ± 8.16**	**0.89 ± 0.11**

**Table 4:** Ten-fold cross-validation classification performance of I-VISTA model best vs. ten random runs.

Fold	Best Accuracy	Accuracy	Sensitivity	Specificity	F1 Score G1	F1 Score G2
1	100	92.72 ± 3.83	100 ± 0.0	86.66 ± 7.02	91.91 ± 3.03	91.91 ± 3.03
2	90.90	79.99 ± 8.35	76.00 ± 18.37	83.33 ± 15.71	80.44 ± 7.66	75.56 ± 10.70
3	81.81	71.81 ± 5.15	58 ± 11.35	83.33 ± 0.0	75.70 ± 2.42	62.96 ± 7.34
4	100	97.27 ± 4.39	100 ± 0.0	95.00 ± 8.05	96.96 ± 4.54	96.96 ± 4.54
5	91.66	69.99 ± 8.04	43.33 ± 16.10	96.66 ± 7.02	74.76 ± 2.5	54.07 ± 6.40
6	100	91.66 ± 3.92	83.33 ± 7.85	100 ± 0.0	91.57 ± 2.19	89.69 ± 3.63
7	83.33	76.66 ± 5.27	91.66 ± 8.78	61.66 ± 15.81	70.16 ± 9.57	79.42 ± 3.43
8	91.66	91.66 ± 0.0	100 ± 0.0	83.33 ± 0.0	90.90 ± 0.0	92.30 ± 0.0
9	100	85.45 ± 6.35	73.33 ± 11.65	100 ± 0.0	85.01 ± 3.34	82.42 ± 4.81
10	100	87.27 ± 10.67	76.66 ± 19.56	100 ± 0.0	87.29 ± 8.74	83.90 ± 12.53

**Table 5:** Average classification performance in ten folds.

Model	Accuracy [95% CI]	Sensitivity (%)	Specificity (%)	AUC [95% CI]
Ref. [30]	81.00 [58.1 94.6]	80.00	81.80	0.89 [0.73 1]
I-VISTA	93.93 [89.7 98.1]	92.66	94.99	0.93 [0.87 0.98]

**Figure A1:**
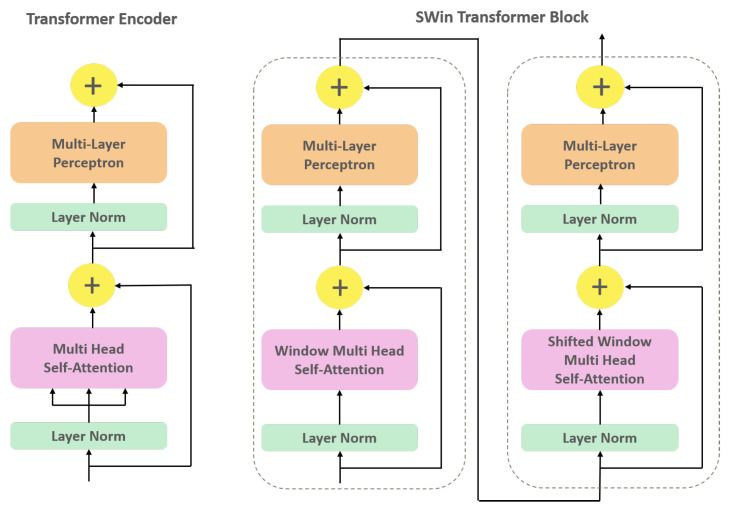
Structure of the original Transformer encoder (**left**) and SWin Transformer block (**right**) highlighting the substitution of the Multi-head Self-Attention (MSA) module with Window-MSA and Shifted Window-MSA.

## Data Availability

The datasets generated and analyzed during the current study are available from the corresponding author on reasonable request.

## Abbreviations

The following abbreviations are used in this manuscript:

AAH	Atypical Adenomatous Hyperplasia
AIS	Adenocarcinoma in Situ
AI	Artificial Intelligence
AUC	Area Under the ROC Curve
CAD	Computer-Aided Detection
CAE	Convolutional Auto-Encoder
CI	Confidence Interval
CCA	Criss-Cross Attention
CNN	Convolutional Neural Network
CT	Computed Tomography
DL	Deep Learning
DT	Decision Tree
FC	Fully Connected
GGN	Ground-Glass Nodule
GMP	Global Max Pooling
HC	Hand-Crafted
IPA	Invasive Pulmonary Adenocarcinoma
k-NN	k-Nearest Neighbors
LASSO	Least Absolute Shrinkage and Selection Operator
LIDC-IDRI	Lung Image Database Consortium and Image Database Resource Initiative
LR	Logistic Regression
LSTM	Long Short-Term Memory
MIA	Minimally Invasive Adenocarcinoma
ML	Machine Learning
MLP	Multi-Layer Perceptron
MPP	Mean of Positive Pixels
MSE	Mean Squared Error
NLP	Natural Language Processing
NSCLC	Non-Small-Cell Lung Carcinoma
PET	Positron Emission Tomography
REB	Research Ethics Board
RF	Random Forest
ROC	Receiver Operating Characteristic
RNN	Recurrent Neural Network
SD	Standard Deviation
SSF	Spatial Scale Filter
SSN	Subsolid Nodule
SVM	Support Vector Machine
SWin	Shifted Window
ViT	Vision Transformer

## Appendix A

In this supplementary document, we will delve into the details of two key components of our I-VISTA model architecture: Transformer Encoder and SWin Transformer. We will begin with an overview of the Transformer encoder architecture, focusing on its self-attention mechanism and how it processes input sequences to capture relationships between different elements. Following this, we will introduce the SWin Transformer, a variant that extends the Transformer architecture by incorporating a hierarchical feature representation and a shifting window mechanism.

### Appendix A.1. Transformer Encoder

The Transformer architecture is established according to the concept of a self-attention mechanism, which computes the relationships between different sequence instances of input. Figure A1 presents a comprehensive illustration of the Transformer encoder architecture. The input data of a Transformer is first tokenized into a sequence of tokens and each token is then embedded into a continuous vector representation. In a self-attention mechanism, the input data is transformed into three different representations, i.e., the Query (*Q*), Key (*K*), and Value (*V*) with dimensions $d_{k}$, $d_{k}$, and $d_{v}$, respectively. These representations are used to compute attention scores, which are then applied to weigh the elements of the input data and generate a weighted representation of the input. Hence, the output is a weighted sum of the values for each token in the sequence, computed as the dot product of the *Q* and *K* vectors for that token. The dot product measures the similarity between the query and each key, and the resulting attention scores represent the relevance of each element in the input data to the current query. The attention function output is computed as follows

(A1) $$Attention\left(Q,K,V\right)=\mathrm{softmax}\left(\frac{QK^{T}}{\sqrt{d_{k}}}\right)V.$$

where superscript ^*T*^ indicates the transpose of a given matrix. For each input element, attention scores can be computed *h* times in parallel with various linear projections implemented each by a different “head” to provide more informative presentations of data in a process known as Multi-head Self-Attention (MSA). The multiple attention scores are then concatenated and processed by a feed-forward neural network to produce the final attention weights. The output of the MSA can be presented as follows

(A2) $$\begin{array}{ccc} MSA(Q,K,V) & \;\;\;\;=\;\;\;\; & \;Concat\left(head_{1},\ldots ,head_{h}\right)W^{O},\\ \mathrm{where}\;\;head_{i} & \;\;\;\;=\;\;\;\; & \;Attention(QW_{i}^{Q},KW_{i}^{K},VW_{i}^{V}). \end{array}$$

where the projections are achieved by parameter matrices $W_{i}^{Q}\in R^{d_{model}\times d_{k}}$, $W_{i}^{K}\in R^{d_{model}\times d_{k}}$, $W_{i}^{V}\in R^{d_{model}\times d_{v}}$, and $W^{O}\in R^{hd_{v}\times d_{model}}$.

The MSA technique allows the model to capture more fine-grained relationships in the data with a quadratic computational complexity with respect to input image size. The MSA output is then added to the input feature maps through a residual connection, which preserves the low-level features of the input. Afterward, the resulting feature maps are normalized using Layer Normalization (LN) and passed on to the next module consisting of a Multi-Layer Perceptron (MLP) and another residual connection. This process is illustrated in Figure A1.

### Appendix A.2. SWin Transformer

SWin Transformer is an advanced variant of Transformer architecture that incorporates hierarchical feature learning and local window-based attention mechanisms. SWin Transformer blocks closely resemble the original Transformer encoder architecture, with the exception of the MSA module (Figure A1). The SWin Transformer block comprises two consecutive Transformer encoder units, each with a unique module replacing the original MSA. The MSA module is modified by utilizing a shifting window mechanism, which allows for computing self-attention within local windows rather than the global attention mechanism used in the original Transformer. The first module is the Window MSA (W-MSA), which computes self-attention within non-overlapping windows of patches, where each window has a size of M×M patches. This modification enables the SWin Transformer to capture local dependencies between patches while simultaneously reducing the computational complexity of the self-attention operation. The W-MSA module has a fixed number of patches within each window throughout the network. Consequently, the SWin Transformer computational complexity scales linearly with input image size. This design ensures that the computational requirements of the SWin Transformer remain consistent, regardless of the input image size. The subsequent Transformer encoder unit includes Shifted Window MSA (SW-MSA) module, which calculates self-attention within shifted W-MSA windows generated by a shift factor of $\frac{M}{2}$. This means that each window is shifted by half its size, allowing the SW-MSA module to capture overlapping features between adjacent windows. The output of a SWin Transformer block can be calculated as

(A3) $$\begin{array}{ccc} {\hat{z}}^{\left(l\right)} & = & W\text{-}\mathrm{MSA}\left(\mathrm{LN}\left(z^{\left(l-1\right)}\right)\right)+z^{\left(l-1\right)},\\ z^{\left(l\right)} & = & \mathrm{MLP}\left(\mathrm{LN}\left({\hat{z}}^{\left(l\right)}\right)\right)+{\hat{z}}^{\left(l\right)},\\ {\hat{z}}^{\left(l+1\right)}\;\;\;\;\;\; & = & \mathrm{SW}\text{-}\mathrm{MSA}\left(\mathrm{LN}\left(z^{\left(l\right)}\right)\right)+z^{\left(l\right)},\\ \mathrm{and}\;\;z^{\left(l+1\right)}\;\;\;\;\;\; & = & \mathrm{MLP}\left(\mathrm{LN}\left({\hat{z}}^{\left(l+1\right)}\right)\right)+{\hat{z}}^{\left(l+1\right)}. \end{array}$$

where ${\hat{z}}^{\left(l\right)}$ and $z^{\left(l\right)}$ are the features produced by W-MSA and MLP modules, respectively.

## References

[1] Thandra K., Barsouk A., Saginala K., Aluru J., Barsouk A. (2021). Epidemiology of lung cancer. Clin. Chest Med..

[2] Thai A., Solomon B., Sequist L., Gainor J., Heist R. (2021). Lung cancer. Lancet.

[3] Cohen J.G., Reymond E., Jankowski A., Brambilla E., Arbib F., Lantuejoul S., Ferretti G.R. (2016). Lung adenocarcinomas: Correlation of computed tomography and pathology findings. Diagn. Interv. Imaging.

[4] Travis W.D., Brambilla E., Nicholson A.G., Yatabe Y., Austin J.H.M., Beasley M.B., Chirieac L.R., Dacic S., Duhig E., Flieder D.B. (2015). The 2015 World Health Organization Classification of Lung Tumors: Impact of Genetic, Clinical and Radiologic Advances Since the 2004 Classification. J. Thorac. Oncol..

[5] Wilkinson A., Lam S. (2021). Lung cancer screening primer: Key information for primary care providers. Can. Fam. Physician.

[6] Amicizia D., Piazza M.F., Marchini F., Astengo M., Grammatico F., Battaglini A., Schenone I., Sticchi C., Lavieri R., Di Silverio B. (2023). Systematic Review of Lung Cancer Screening: Advancements and Strategies for Implementation. Healthcare.

[7] Jin H., Yu C., Gong Z., Zheng R., Zhao Y., Fu Q. (2023). Machine learning techniques for pulmonary nodule computer-aided diagnosis using CT images: A systematic review. Biomed. Signal Process. Control.

[8] Kuhn E., Morbini P., Cancellieri A., Damiani S., Cavazza A., Comin C.E. (2018). Adenocarcinoma classification: Patterns and prognosis. Pathol.-J. Ital. Soc. Anat. Pathol. Diagn. Cytopathol..

[9] Zhang Y., Ma X., Shen X., Wang S., Li Y., Hu H., Chen H. (2022). Surgery for pre- and minimally invasive lung adenocarcinoma. J. Thorac. Cardiovasc. Surg..

[10] Fu F., Chen Z., Chen H. (2022). Treating lung cancer: Defining surgical curative time window. Cell Res..

[11] Wang L. (2022). Deep Learning Techniques to Diagnose Lung Cancer. Cancers.

[12] Christie J.R., Lang P., Zelko L.M., Palma D.A., Abdelrazek M., Mattonen S.A. (2020). Artificial Intelligence in Lung Cancer: Bridging the Gap Between Computational Power and Clinical Decision-Making. Can. Assoc. Radiol. J..

[13] Afshar P., Mohammadi A., Plataniotis K.N., Oikonomou A., Benali H. (2018). From Handcrafted to Deep-Learning-Based Cancer Radiomics: Challenges and opportunities. IEEE Signal Process. Mag..

[14] Feng H., Shi G., Xu Q., Ren J., Wang L., Cai X. (2023). Radiomics-based analysis of CT imaging for the preoperative prediction of invasiveness in pure ground-glass nodule lung adenocarcinomas. Insights Imaging.

[15] Heidarian S., Afshar P., Enshaei N., Naderkhani F., Rafiee M.J., Babaki Fard F., Samimi K., Atashzar S.F., Oikonomou A., Plataniotis K.N. (2021). COVID-FACT: A Fully-Automated Capsule Network-Based Framework for Identification of COVID-19 Cases from Chest CT Scans. Front. Artif. Intell..

[16] Liu S., Xie Y., Jirapatnakul A., Reeves A. (2017). Pulmonary nodule classification in lung cancer screening with three-dimensional convolutional neural networks. J. Med. Imaging.

[17] Wang F., Wang C.-L., Yi Y.-Q., Zhang T., Zhong Y., Zhu J.-J., Li H., Yang G., Yu T.-F., Xu H. (2023). Comparison and fusion prediction model for lung adenocarcinoma with micropapillary and solid pattern using clinicoradiographic, radiomics and deep learning features. Sci. Rep..

[18] Vaswani A., Shazeer N., Parmar N., Uszkoreit J., Jones L., Gomez A.N., Kaiser L., Polosukhin I. Attention is All you Need. Proceedings of the Advances in Neural Information Processing Systems.

[19] Shao T., Guo Y., Chen H., Hao Z. (2019). Transformer-Based Neural Network for Answer Selection in Question Answering. IEEE Access.

[20] Liu H., Chen W. (2021). Re-Transformer: A Self-Attention Based Model for Machine Translation. Procedia Comput. Sci..

[21] Rodrawangpai B., Daungjaiboon W. (2022). Improving text classification with transformers and layer normalization. Mach. Learn. Appl..

[22] Dosovitskiy A., Beyer L., Kolesnikov A., Weissenborn D., Zhai X., Unterthiner T., Dehghani M., Minderer M., Heigold G., Gelly S. (2020). An Image is Worth 16 × 16 Words: Transformers for Image Recognition at Scale. arXiv.

[23] He K., Gan C., Li Z., Rekik I., Yin Z., Ji W., Gao Y., Wang Q., Zhang J., Shen D. (2023). Transformers in medical image analysis. Intell. Med..

[24] Shamshad F., Khan S., Zamir S.W., Khan M.H., Hayat M., Khan F.S., Fu H. (2023). Transformers in medical imaging: A survey. Med. Image Anal..

[25] Barbouchi K., El Hamdi D., Elouedi I., Ben Aïcha T., Kacem Echi A., Slim I. (2022). A transformer-based deep neural network for detection and classification of lung cancer via PET/CT images. Int. J. Imaging Syst. Technol..

[26] Armato S.G., McLennan G., Bidaut L., McNitt-Gray M.F., Meyer C.R., Reeves A.P., Zhao B., Aberle D.R., Henschke C.I., Hoffman E.A. (2011). The Lung Image Database Consortium (LIDC) and Image Database Resource Initiative (IDRI): A completed reference database of lung nodules on CT scans. Med. Phys..

[27] Sun R., Pang Y., Li W. (2023). Efficient Lung Cancer Image Classification and Segmentation Algorithm Based on an Improved Swin Transformer. Electronics.

[28] Mkindu H., Wu L., Zhao Y. (2023). Lung nodule detection in chest CT images based on vision transformer network with Bayesian optimization. Biomed. Signal Process. Control.

[29] Mkindu H., Wu L., Zhao Y. (2023). 3D multi-scale vision transformer for lung nodule detection in chest CT images. Signal Image Video Process..

[30] Oikonomou A., Salazar P., Zhang Y., Hwang D.M., Petersen A., Dmytriw A.A., Paul N.S., Nguyen E.T. (2019). Histogram-based models on non-thin section chest CT predict invasiveness of primary lung adenocarcinoma subsolid nodules. Sci. Rep..

[31] Andersen M.B., Harders S.W., Ganeshan B., Thygesen J., Torp Madsen H.H., Rasmussen F. (2016). CT texture analysis can help differentiate between malignant and benign lymph nodes in the mediastinum in patients suspected for lung cancer. Acta Radiol..

[32] Zerunian M., Caruso D., Zucchelli A., Polici M., Capalbo C., Filetti M., Mazzuca F., Marchetti P., Laghi A. (2021). CT based radiomic approach on first line pembrolizumab in lung cancer. Sci. Rep..

[33] Ganeshan B., Goh V., Mandeville H.C., Ng Q.S., Hoskin P.J., Miles K.A. (2013). Non-small cell lung cancer: Histopathologic correlates for texture parameters at CT. Radiology.

[34] Hofmanninger J., Prayer F., Röhrich S., Prosch H., Langs G. (2020). Automatic lung segmentation in routine imaging is primarily a data diversity problem, not a methodology problem. Eur. Radiol. Exp..

[35] Ronneberger O., Fischer P., Brox T. (2015). U-Net: Convolutional Networks for Biomedical Image Segmentation. International Conference on Medical Image Computing and Computer-Assisted Intervention.

[36] Walsh J., Othmani A., Jain M., Dev S. (2022). Using U-Net network for efficient brain tumor segmentation in MRI images. Healthc. Anal..

[37] Liu W., Luo J., Yang Y., Wang W., Deng J., Yu L. (2022). Automatic lung segmentation in chest X-ray images using improved U-Net. Sci. Rep..

[38] Ayalew Y., Fante K., Mohammed M. (2021). Modified U-Net for liver cancer segmentation from computed tomography images with a new class balancing method. BMC Biomed. Eng..

[39] Liu Z., Lin Y., Cao Y., Hu H., Wei Y., Zhang Z., Lin S., Guo B. Swin transformer: Hierarchical vision transformer using shifted windows. Proceedings of the IEEE/CVF International Conference on Computer Vision.

[40] Zhang L., Wen Y. A transformer-based framework for automatic COVID19 diagnosis in chest CTs. Proceedings of the IEEE/CVF International Conference on Computer Vision Workshops.

[41] Deng J., Dong W., Socher R., Li L.-J., Li K., Fei-Fei L. ImageNet: A large-scale hierarchical image database. Proceedings of the IEEE Conference on Computer Vision and Pattern Recognition.

[42] Masci J., Meier U., Ciresan D., Schmidhuber J. (2011). Stacked Convolutional Auto-Encoders for Hierarchical Feature Extraction. International Conference on Artificial Neural Networks.

[43] Miles K., Ganeshan B., Hayball M. (2013). CT texture analysis using the filtration-histogram method: What do the measurements mean?. Cancer Imaging.

[44] Huang Z., Wang X., Huang L., Huang C., Wei Y., Liu W. CCNet: Criss-Cross Attention for Semantic Segmentation. Proceedings of the IEEE/CVF International Conference on Computer Vision.

[45] Wang H., Zhu H., Ding L., Yang K. (2023). A diagnostic classification of lung nodules using multiple-scale residual network. Sci. Rep..

[46] Sun Y., Li C., Jin L., Gao P., Zhao W., Ma W., Tan M., Wu W., Duan S., Shan Y. (2020). Radiomics for lung adenocarcinoma manifesting as pure ground-glass nodules: Invasive prediction. Eur. Radiol..

[47] Yang Y., Tan M., Ma W., Duan S., Huang X., Jin L., Tang L., Li M. (2022). Preoperative prediction of the degree of differentiation of lung adenocarcinoma presenting as sub-solid or solid nodules with a radiomics nomogram. Clin. Radiol..

[48] Feng B., Chen X., Chen Y., Li Z., Hao Y., Zhang C., Li R., Liao Y., Zhang X., Huang Y. (2019). Differentiating minimally invasive and invasive adenocarcinomas in patients with solitary sub-solid pulmonary nodules with a radiomics nomogram. Clin. Radiol..

[49] Afshar P., Oikonomou A., Naderkhani F., Tyrrell P.N., Plataniotis K.N., Farahani K., Mohammadi A. (2020). 3D-MCN: A 3D Multi-scale Capsule Network for Lung Nodule Malignancy Prediction. Sci. Rep..

[50] Zhou J., Hu B., Feng W., Zhang Z., Fu X., Shao H., Wang H., Jin L., Ai S., Ji Y. (2023). An ensemble deep learning model for risk stratification of invasive lung adenocarcinoma using thin-slice CT. NPJ Digit. Med..

[51] Zhang Y., Qu H., Tian Y., Na F., Yan J., Wu Y., Cui X., Li Z., Zhao M. (2023). PB-LNet: A model for predicting pathological subtypes of pulmonary nodules on CT images. BMC Cancer.

[52] Wang X., Zhang L., Yang X., Tang L., Zhao J., Chen G., Li X., Yan S., Li S., Yang Y. (2020). Deep learning combined with radiomics may optimize the prediction in differentiating high-grade lung adenocarcinomas in ground glass opacity lesions on CT scans. Eur. J. Radiol..

